# Analysis of the complete lambda light chain germline usage in patients with AL amyloidosis and dominant heart or kidney involvement

**DOI:** 10.1371/journal.pone.0264407

**Published:** 2022-02-25

**Authors:** Natalie Berghaus, Sarah Schreiner, Martin Granzow, Carsten Müller-Tidow, Ute Hegenbart, Stefan O. Schönland, Stefanie Huhn

**Affiliations:** 1 Medical Department V, Amyloidosis Center, Heidelberg University Hospital, Heidelberg, Germany; 2 Institute of Human Genetics, Heidelberg University Hospital, Heidelberg, Germany; 3 Medical Department V, Heidelberg University Hospital, Heidelberg, Germany; 4 National Centre for Tumor Diseases (NCT), Heidelberg, Germany; 5 Medical Department V, Section of Multiple Myeloma, Heidelberg University Hospital, Heidelberg, Germany; Chang Gung University, TAIWAN

## Abstract

Light chain amyloidosis is one of the most common forms of systemic amyloidosis. The disease is caused by the misfolding and aggregation of immunoglobulin light chains to insoluble fibrils. These fibrils can deposit in different tissues and organs such as heart and kidney and cause organ impairments that define the clinical presentation. In this study, we present an overview of IGLV-IGLJ and IGLC germline utilization in 85 patients classified in three clinically important subgroups with dominant cardiac, renal as well as cardiac and renal involvement. We found that IGLV3 was the most frequently detected IGLV-family in patients with dominant cardiac involvement, whereas in renal patients IGLV1 were most frequently identified. For patients with dominant heart and kidney involvement IGLV6 was the most frequently detected IGLV-family. In more detailed analysis IGLV3-21 was observed as the most dominant IGLV-subfamily for patients with dominant heart involvement and IGLV1-44 as the most frequent IGLV-subfamily in the group of patients with dominant kidney involvement. For patients with dominant heart and kidney involvement IGLV6-57 was the most frequently detected IGLV-subfamily. Additionally, we were able to show an exclusive linkage between IGLJ1 and IGLC1 as well as between IGLJ2 and IGLC2 in the fully assembled IGL mRNA.

## Introduction

With an incidence rate of 3–5 patients per million, light chain amyloidosis (AL) is the most common form of systemic amyloidosis (70%) [1]. It is classified as a clonal plasma cell (PC) disease characterized by an overproduction of free light chains (LCs), which misfold, aggregate and deposit in tissue in form of amyloid fibrils [2]. These fibrils and their precursors have a devastating effect on the organ function and make this rare disease life threatening. Still, it remains unknown why some AL patients show a distinct organ tropism to one organ such as heart or kidney. Cardiac involvement can be observed in about 80% of the patients, and as the second most common form renal involvement could be observed in about 70% [2].

In the last decades, several research groups have tried to uncover the causes of fibril formation of LCs using different approaches and it was possible to solve the cryo-EM structures of ex vivo fibrils [3, 4]. Furthermore, specific mutations could be identified facilitating fibril formation of one truncated LC [5]. However, most studies describe only one or few specific LCs/cases, so that it is still rather difficult to state consequences and imply mechanisms for the disease in general.

In this study we aimed to enhance the knowledge about the germline usage of the V-segment in three clinically distinct subgroups of AL patients with lambda LC restriction and with dominant heart, dominant kidney or dominant heart and kidney involvement. This is based on the assumption that the use of certain IGLV segments poses a risk for the development of amyloidosis and might explain organ tropism [6–10]. Moreover, we analyzed not only the IGLV composition, but also the complete lambda LC and its IGLV-IGLJ-IGLC composition. In contrast to published studies, we performed our analysis on CD138+ positive selected bone marrow (BM) PC, which allowed us to obtain a pure sample of tumor cells. CD138 is described as an antigen highly expressed on malignant PC in peripheral blood and BM of patients with monoclonal gammopathies such as AL and Multiple Myeloma (MM) [11–14]. In the diagnostic work up of those patients CD138+ cells provide the basis for interphase FISH diagnostics with prognostic impact into AL and MM [15]. Data from an extensive FISH-Panel provide us further information about the clonality of the samples [16].

For sequencing, we used an adapted primer-set which designed for highly sensitive minimal residue disease diagnostics in MM [17, 18]. These approaches allowed us to find the most dominant clone in a heterogenic PC population.

## Materials and methods

### Patient population and clinical data

We included patients which were seen between March 2019 and March 2021 at the Amyloidosis Centre of the University Hospital Heidelberg. This study was approved by the Ethics Committee of the University of Heidelberg (S-123/2006, last time renewed 12.06.2018) and followed the Helsinki guidelines for research of human subjects. Data was collected for FISH analysis and CD138+ sorting (Table 1). Clinical parameters were collected and analyzed on the basis of the clinical patient reports (Table 2). The vast majority of patients were sampled at time of diagnosis at the Amyloidosis Centre. Only seven patients underwent BM aspiration at a state of relapse or progress of the clonal disease. We have indicated patients having “dominant heart involvement”, “dominant renal involvement” or “dominant heart and renal involvement” if they had no other clinically relevant organ manifestation.

**Table 1:** Comparison of the CD138+ cell-sorting and of the main clone in the FISH-analysis.

	Dominant heart involvement	Dominant kidney involvement	Dominant heart and kidney involvement
	n = 47	n = 25	n = 13
Mean BM aspirate volume [ml] (range)	54 (33–84)	53 (25–90)	51 (10–65)
Mean percentage of CD138+ PC in the sample (range)	0.9 (0–4.5)	0.7 (0–5.2)	1.5 (0.1–12.2)
Number of cell sorts with no harvested CD138+ PC (%)	4 (8.5)	1 (4)	0
Mean percentage of the main clone in the FiSH analysis (range)	85 (43–98)	72 (27–97)	82 (63–96)

Divided in patients with dominant heart (n = 47), kidney (n = 25) and heart and kidney (n = 13) involvement, BM = Bone marrow, PC = plasmacell.

**Table 2:** Characteristics of 85 patients with AL amyloidosis were included in this study.

Characteristic	Dominant heart involvement	Dominant kidney involvement	Dominant heart and kidney involvement
	n = 47	n = 25	n = 13
Mean age, [y] (range)	65 (34–86)	59 (38–75)	63 (51–83)
Sex female/male	17/30	9/16	6/7
Mean Karnofsky-Index (range)	82 (50–100)	91 (60–100)	90 (80–100)
New-diagnosis, n	43	22	13
Relapse/late relapse/progress, n	4	3	0
Organ involvement [%]			
Heart	100	16	100
Kidney	6	100	100
Liver	0	0	0
Lung	0	0	0
ANS	11	4	8
PNS	4	4	8
GI	0	4	15
ST	40	4	38
Mean dFLC at diagnosis [mg/l] (range)	559 (-253-1778)	65 (-14-284)	393 (67–2065)
Mean secretion lambda LC urine [mg/d] (range)	185 (0–1490)	255 (11–3630)	109 (15–423)
Median plasma cell infiltration [%] (range)	14 (0.5–56)	8 (2–20)	17 (3–55)
M-Gradient, n (%)	17 (36)	12 (48)	6 (46)
T(11;14) n (%)	18 (38)	16 (64)	8 (62)
Circulating monoclonal protein in the serum, n (%)	19 (40)	15 (60)	8 (62)
IgG, n (%)	16 (34)	10 (40)	4 (31)
IgA, n (%)	2 (4)	4 (16)	4 (31)
IgM, n (%)	0	1 (4)	0
IgG and IgD, n (%)	1 (2)	0	0
Kidney parameters			
Mean proteinuria [g/d] (SD)	0.7 (0.9)	6.3 (4.4)	4.7 (3.4)
Mean GFR CKD-EPI (SD)	68 (21)	74 (30)	74 (25)
Heart parameters			
Mean NT-proBNP, serum [ng/l], (SD)	10458 (11439)	910 (2570)	4453 (4376)
Mean TNT [μg/l] (SD)	0.21 (0.2)	0.03 (0.04)	0.08 (0.1)
Mean AP [U/l] (SD)	106 (321)	74 (19)	80 (131)

Divided in patients with dominant heart (n = 47), kidney (n = 25) and heart and kidney (n = 13) involvement. ANS = autonomic nervous system, PNS = peripheral nervous system, GI = gastrointestinal tract, SI = soft tissue.

### Bone marrow sample preparation

10–90 ml BM aspirates were collected from patients with lambda AL Amyloidosis with dominant heart, kidney or heart and kidney involvement. Mononuclear cells were isolated by density gradient centrifugation (Ficoll Paque). After washing the cells, a red cell lysis was performed. CD138+ PC were extracted using magnetic beads-based positive selection (STEMCELL Technologies, Vancouver Canada). Depending on the cell count of the positive fraction 60 000–30 0000 PC were used for FISH analysis. The highest measured percentage of a genetic aberration in the FISH result was defined as the proportion of the main clone [19]. The remaining CD138+ PC were stored in guanidinium thiocyanate (GTC)-buffer with beta-mercaptoenthanol at -80°C for RNA stabilization.

### Isolation of RNA, reverse transcriptase

The isolation of genomic DNA and RNA was performed using the „AllPrep DNA/RNA/Protein Mini Kit”(Qiagen, Venlo, Netherlands), according the manufactures instructions. To homogenize the lysate a QIAshredder was used and due to the storage of the samples in GTC-buffer no additional RLT-buffer with β-mercaptoethanol was added. To eluate the total RNA, 15 μl RNAse free water was added twice and centrifuged to obtain a final volume of 30 μl total RNA. The reverse transcriptase reaction was performed with the High Capacity cDNA Reverse Transcription Kit with RNAse Inhibitor and Oligo dT18-Primer (Thermo Scientific, Waltham, Massachusetts, USA).

### Light chain amplification and sequencing

The polymerase-chain-reaction (PCR) was performed following the published protocol [17]. Final PCR volumes were 25 μl with 1 μl template, according to the recommended protocol of AmpliTaq Gold DNA Polymerase (Thermo Scientific). The forward primers were adapted from Huhn S. [17]. One additional forward primer was designed specifically for detecting IGLV6 in a single-plex setup (S1 Table). A reverse primer was designed based on the most common IGLC-segments (S1 Table). The result of the PCR-reaction was checked with an analytic gel-electrophoresis. PCR-products were cleaned with the „High Pure PCR-Product Purification Kit”(Roche, Basel, Switzerland) according to the manufactures instructions. The elution-step was performed with 2 times 15 μl (2x 13 000g for 1 min) instead of 50–100 μl elution-buffer. Together with the corresponding primers the samples were sent to Eurofins/GATC and analyzed through Sanger Sequencing. The samples were adjusted to a final DNA concentration according to the guidelines.

### Sequence alignment and statistical analysis

For obtaining a sequence, the ab1-Files from Eurofins were analyzed. For IGLV- and IGLJ-gen family assignment VBase2 [20] was used. For translation Expasy [21] were used and the AS-sequences were analyzed with Ensembl BLAST [22]. In the text IGLJ1*01 (Gene ID: 28833) is defined as IGLJ1. IGLJ2*01 and IGLJ3*01 (Gene ID: 28832) show the same cDNA and AS-Sequence and are therefore defined as IGLJ2.IGLJ3*02 (Gene ID: 28831) is defined as IGLJ3. Based on the Ensembl BLAST results IGLC-assignment was done. The sequences with the highest similarity score were aligned to the patient specific sequences by MEGA [23] and Clustal Omega [24] to identify unique variations. The quality of the sequences and the confidence of VJC-family assignments were rated based on the count of insecurities resulting from overlapping nucleotide signals in the sequencing. If more than 10 AS (about 6% of the LC sequence) in the translation could not be clearly identified a resequencing with an IGLV-family specific primer was performed. We stated a case as “not identified”, when it was not possible to make a clear family assignment at cDNA- and AS-level. In addition, data from AL-Base [25] was used for comparison purposes.

## Results

### Patient population and bone marrow aspiration

BM was collected from 101 patients during the specified period. In five cases we were not able to detect any CD138+ PC. However, these were also used for further LC analysis, since the detection limit of the cell count is 2,5E+04 CD138+ PC and it is assumed that sufficient cells are still available for further molecular biological analysis. Through FISH diagnostics, we can obtain information about the clonality of the samples (Table 1). In 16 cases, there were not enough CD138+ PC for further analysis, after cells have been used for FISH analysis. In the final group of the 85 patients, 47 (55%) patients were classified as having dominant heart disease, 25 (29%) patients having dominant kidney disease and 13 (15%) patients having dominant kidney and heart disease (S1 Fig).

The clinical characteristics of the different cohorts are listed in Table 2. The population with dominant heart involvement (14%) and the one with dominant heart and kidney involvement (17%) showed a two times higher value of median PC infiltration compared to the population with dominant kidney involvement (8%). The average value of the difference between involved and uninvolved serum immunoglobulin free light chain levels (dFLC) at diagnosis of the population with dominant heart involvement (559 mg/l) were over eight times higher compared to the group with dominant kidney involvement (65 mg/l). No other organ involvement was detected for patients with dominant kidney involvement in 19 cases (76%), for patients with dominant heart involvement in 27 cases (57%) and for patients with heart and kidney involvement in 6 cases (46%). Additional soft tissue involvement was observed in about 40% of the patients with dominant cardiac manifestation, in 38% of the patients with dominant cardiac and kidney manifestation and in 4% of the patients with dominant renal involvement.

### Family and germline usage

**Fig 1:**
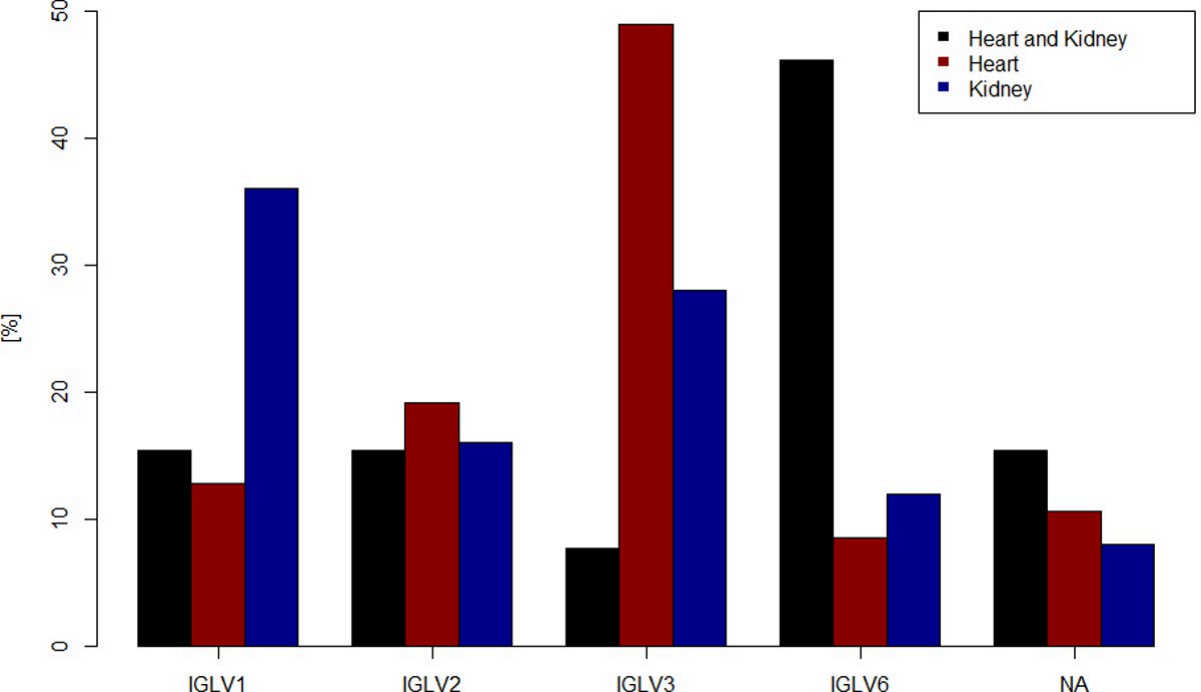
Comparison of the Vλ-family expression. Dominant heart and kidney involvement = Heart and Kidney (black), dominant heart involvement = Heart (red), dominant kidney involvement = Kidney (blue).

For patients with dominant heart involvement the IGLV3-family was assigned in 49% of the cases, for the patients with dominant kidney involvement in 28% and for patients with dominant heart and kidney involvement only in 8%. Thirty-six percent of the sequences from patients with dominant kidney involvement where attributed to IGLV1 (dominant heart involvement 13%, dominant heart and kidney involvement 15%). Concerning the frequency of assignment to IGLV6, a difference was also detected in terms of organ manifestation. For dominant cardiac disease, IGLV6 was assigned in 9%, for dominant renal disease in 12% of the cases, but for dominant heart and kidney involvement in 46% of the cases (Fig 1 and S2 Table).

Regarding the clonality of the samples, no significant difference was found between the IGLV-families. For samples classified in the IGLV1-family, the average percentage of the main clone was 78% (SD: 16%), for IGLV2-samples 85% (SD: 10%), for IGLV3-samples 85% (SD: 15%) and for samples of the IGLV6-family 74% (SD: 17%).

In nine (11%) cases, no clear assignment could be done to an IGLV-gene or family. These included five (11%) cases for dominant heart, two (8%) cases for dominant kidney and two (15%) cases for dominant heart and kidney involvement. These nine samples had an average clonality of 71% (SD: 23%), which was slightly lower than the values of the respective subgroups. In one of these nine cases (dominant heart involvement) the PCR with the multiplex-primer set and the PCR with the IGLV6 specific primer achieved sequences with nearly the same number of blurs. This sequence was not assigned to any IGLV-family in the evaluation and defined as non-evaluable.

**Fig 2:**
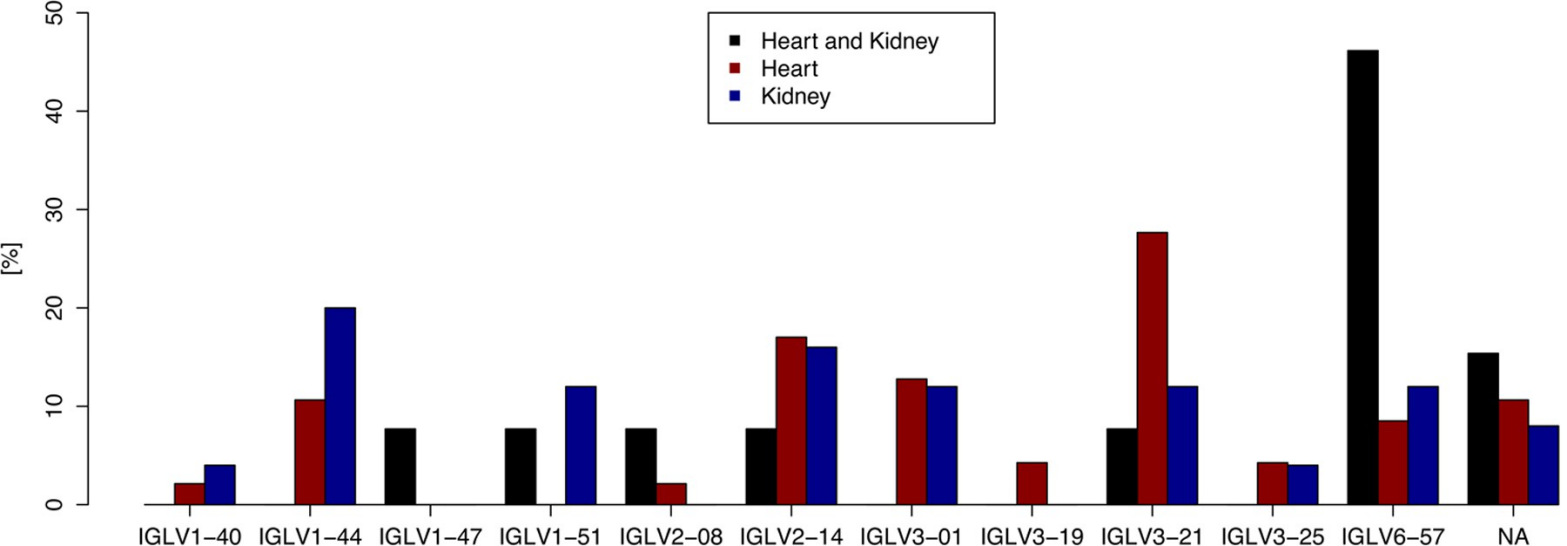
Comparison of the usage of the Vλ germline repertoire. Dominant heart and kidney involvement = Heart and Kidney (black), dominant heart involvement = Heart (red), dominant kidney involvement = Kidney (blue).

For patients with dominant heart disease, IGLV3-21 was estimated as the most frequent IGLV-family (28%), while IGLV2-14 was the second most frequent detected (17%), and IGLV3-1 with 13% (Fig 2 and S3 Table). For the group of patients with dominant kidney involvement IGLV1-44 was detected as the most frequent IGLV-family (20%). It was followed by IGLV2-14 with 16% and IGLV1-51, IGLV3-1, IGLV3-21 and IGLV6-57 with 12%. For patients with dominant heart and kidney involvement IGLV6-57 was with 46% the most frequently detected IGLV-family, any other families were detected in 8%. The IGLV1-47 family was detected only once in a patient with dominant heart and kidney disease. The IGLV3-19 family could only be detected twice, both patients showed a dominant cardiac involvement. No significant difference between clonality was detected in FISH between the two most dominant IGLV-subgroups. For samples of the family IGLV3-21 the main clone could be detected with an average of 86% (SD: 13%), for IGLV6-57 with an average of 74% (SD: 17%).

We also analyzed the IGLJ and IGLC germline usage (Table 3) in the different groups of patients. Overall IGLJ2 (34%) and IGLJ3 (35%) were most frequently detected in the samples. For the C-segment IGLC2 (53%) was observed with the highest frequency in all three subgroups followed by IGLC3 (22%) and IGLC1 (16%). No significant difference was found between the subgroups in terms of frequency.

**Table 3:** Comparison of the usage of the IGLJ and IGLC germline.

	IGLJ1	IGLJ2	IGLJ3	IGLJ2/ IGLJ3	NA	IGLV1	IGLC2	IGLC3	IGLC2/ IGLC3	NA
Heart [%] n = 47	19	30	32	15	4	19	57	19	2	2
Kidney [%] n = 25	20	36	36	8	0	16	48	24	8	4
Heart and Kidney [%] n = 13	8	46	46	0	0	8	46	31	8	8

In the analysis of preferential joining of specific IGLJ- and IGLC-family members, our dataset showed that IGLJ1 and IGLJ2 were exclusively joined to IGLC1 and IGLC2, respectively. In contrast, IGLJ3 was found joined to IGLC2 (40%) or IGLC3 (53%) without any relevant preference for one of the two while joining with IGLC1 has not been detected.

Finally, we also analyzed the composition of the IGLV-IGLJ-IGLC germline usage (Table 4). Due to the low sample size compared to the number of possible triplet combinations, it was not possible to make clear statements about individual combinations. However, certain trends can be identified about preferential joining of individual IGLV-families to JC-segments. In six out of eight IGLV3-1-cases IGLV3-1 was found joined with IGLJ1 and IGLC1. Of note the LCs from patients with dominant kidney involvement and IGLV3-1 were found exclusively joined to IGLJ1-IGLC1. Also, IGLV2-14 was found connected with IGLJ1 and IGLC1 in four out of eight cases from patients with dominant heart involvement connected. In case of IGLV6-57 LCs in 46% an assembly with IGLJ2 and IGLC2 and in 31% an assembly with IGLJ2 and IGLC2 was found. For IGLV3-21 and IGLV6-57 no connection with IGLJ1 and IGLC was detected.

**Table 4:** Analysis of the usage of Jλ germline und Cλ germline repertoire depending on the used Vλ germline.

	n	IGLJ1 IGLC1	IGLJ2 IGLC2	IGLJ3 IGLC2	IGLJ3 IGLC3	IGLJ2/ IGL3 IGLC2	IGLJ2/ IGLJ3 IGLC3	NA
**IGLV1-44 (%)**	**10**	**1 (10)**	**2 (20)**	**3 (30)**	**2 (20)**	**1 (10)**	**0**	**1 (10)**
Heart	5	1	0	2	1	0	0	1
Kidney	5	0	2	1	1	1	0	0
Heart and kidney	0	0	0	0	0	0	0	0
**IGLV2-14 (%)**	**13**	**4 (31)**	**4(31)**	**0**	**3 (23)**	**0**	**1 (8)**	**1 (8)**
Heart	8	4	1	0	2	0	1	0
Kidney	4	0	2	0	1	0	0	1
Heart and kidney	1	0	1	0	0	0	0	0
**IGLV3-1 (%)**	**8**	**6 (75)**	**1 (13)**	**1 (13)**	**0**	**0**	**0**	**0**
Heart	5	3	1	1	0	0	0	0
Kidney	3	3	0	0	0	0	0	0
Heart and kidney	0	0	0	0	0	0	0	0
**IGLV3-21 (%)**	**18**	**0**	**6 (33)**	**1(6)**	**4 (22)**	**3 (17)**	**1 (6)**	**1 (6)**
Heart	13	0	4	1	4	2	1	1
Kidney	3	0	1	0	0	1	0	1
Heart and kidney	1	0	1	0	0	0	0	0
**IGLV6-57 (%)**	**13**	**0**	**6 (46)**	**3 (23)**	**4 (31)**	**0**	**0**	**0**
Heart	4	0	2	2	0	0	0	0
Kidney	3	0	1	0	2	0	0	0
Heart and kidney	6	0	3	1	2	0	0	0

Joining partner for IGLV-families found in less than 5 cases are not shown (IGLV1-40, n = 2; IGLV1-47, n = 1; IGLV1-51, n = 4; IGLV2-8, n = 2; IGLV3-19, n = 2; IGLV3-25, n = 3). Dominant heart and kidney involvement = Heart and Kidney (black), dominant heart involvement = Heart (red), dominant kidney involvement = Kidney (blue).

## Discussion

Several studies have already been conducted on the use of IGLV-germline segments in AL amyloidosis [6–10, 26] (Table 5). For this purpose, results from mainly mass spectrometry (MS) analyses and cDNA analyses using cloning techniques were used. Our study is the first that used BM cells selected for CD138+ before sequencing. This method has the advantage of obtaining a sample with a very high purity of tumor cells, as on average only less than 1% CD138+ cells could be detected in unsorted material. The enrichment of CD138+ fraction significantly reduces background signals in sequencing and allows analysis of IG recombination of the malignant PCs with fewer disturbance of germline signals. Furthermore, the FISH analysis allows us to draw conclusions about the PC clonality of the samples. On average, about 81% of all cells analyzed belonged to the main clone. There was no significant difference between the two subgroups with dominant organ manifestation and between the individual IGLV-families in terms of clonality, although the PC clone of the patients with dominant heart involvement and dominant heart and kidney involvement were bigger (S3 Fig). We also investigated a potential correlation between the dFLC values at diagnosis and the dominant IGLV-subfamilies. Also, a potential correlation between the dFLC values at diagnosis and the dominant IGLV-subfamilies was investigated. No significant difference could be found between the subgroups (pairwise comparison among all groups p-value >0.2) (S4 Fig).

**Table 5:** Comparison between previously published data and data from this study.

Publication	Method and biomaterial	Number of λ patients/ IGLV identified	IGLV-family complete cohort	IGLV-family dominant heart involvement	IGLV-family dominant kidney involvement
This work	cDNA sequencing;	85 / 89%	-	1. IGLV3-21	1. IGLV1-44
	Bone marrow mononuclear cells;			2. IGLV2-14	2. IGLV2-14
	CD138+ cell sorting			3. IGLV3-1	3. IGLV1-51
					3. IGLV3-1
					3. IGLV3-21
					3. IGLV6-57
Comenzo et al. 2001, Blood	cDNA sequencing through cloning technique;	48 / 72% (κ and λ)	1. IGLV6-57	27% of the complete cohort	54% of the complete cohort
			2. IGLV1-44		
	Bone marrow (after red blood cell lysis)				
			3. IGLV3-1	1. IGLV1-44	1. IGLV6-57
				2. IGLV3-1	2. IGLV3-1
				2. IGLV2-14	2. IGLV1-40
				3. IGLV6-57	2. IGLV1-51
					2. IGLV2-14
Perfetti et al. 2002, Blood	cDNA sequencing through cloning technique;	55 / 100%	1. IGLV3-1	-	IGLV6-57
			2. IGLV6-57		
			3. IGLV3-21		
	Bone marrow, mononuclear cells				
Abraham et al.2003, Blood	cDNA sequencing;	32 / 100%	1. IGLV3-1	1. IGLV2	1. IGLV6
	Bone marrow, mononuclear cells		1. IGLV6-57	2. IGLV1	2. IGLV1
			3. IGLV2-23	3. IGLV3	2. IGLV2
				3. IGLV6	
Perfetti et al. 2012, Blood	cDNA sequencing through cloning technique;	99 / 100%	1. IGLV6-57	56% of the complete cohort	-
			2. IGLV3-1		
	Bone marrow, mononuclear cells		3. IGLV2-14		
				1. IGLV1-44	
				2. IGLV3-21	
				3. IGLV2-14	
Kourelis et al. 2017, Blood	LC-MS and cDNA sequencing;	514 / 82% (κ and λ)	1. IGLV6-57	1. IGLV3-19	1. IGLV6-57
			2. IGLV3-1	2. IGLV1-44	2. IGLV1-40
	solid tissue specimen, fat aspirate specimen for LC-MS; Bone marrow for cDNA Sequencing (N = 30)		3. IGLV2-14	3. IGLV1-40	3. IGLV2-14
Sidana et al. 2021, Blood advanced	LC-MS;	297 / 98% (IgM + κ and λ)	1. IGLV6-57	-	IGLV6-57
	Fat aspirates, formalin fixed paraffin embedded tissue biopsy specimen		2. IGLV3-1		
			3. IGLV1-44		
			3. IGLV2-14		

Another interesting difference between the three subgroups is that about 40% of the patients with dominant cardiac manifestation presented soft tissue involvement, whereas this was only the case in 4% of the patients with dominant renal involvement, but in 33% of the patients with dominant heart and kidney involvement. This indicates that involvement of the heart also favors soft tissue infestation.

For the interpretation of our study and for comparison to the already published data it must be considered that the source of biomaterial, the measurement methods and the composition of the cohort play a crucial role and that the presented results hold true under the specific conditions described above. By a clear clinical classification of the groups and our molecular biological approach of the CD138+ positive selection and the multiplex primer set we tried to exclude as many interfering factors as possible. In general, our IGLV-family identification success rate of 89% is within the range of other cDNA based published data sets (72%-100%).

If we hypothesize that different organ tropisms favor certain IGLV germline usage, this is an essential point to compare with existing data. In the study of Perfetti et al. in 2002, a singular organ infestation could only be defined in 38%, a double organ infestation was present in 42% [8]. In 2012, similar percentages were reported for the cohort with dominant heart involvement [8, 9]. In the study by Comenzo et al., an involvement of 1–2 organs was defined in 68% and an involvement of three or more organs in 32% [6]. In 2003 Abraham et al. reported nearly the same values as Comenzo and Perfetti, 63% of the patients showed an involvement of two or more organs and only 35% showed only involvement of one organ [7]. Kourelis et al. defined additionally groups of isolated heart and kidney infestation without additional organ manifestation, but they showed no significant differences in IGLV gene usage compared to a group without isolated heart or kidney involvement [10].

In contrast to all six compared publications, in which IGLV6-57 was identified among the top three most common IGLV-families and especially for dominant renal involvement, our results indicate that this may be only a characteristic of patients with dominant heart and kidney involvement. We were able to detect this family in 46% of this cohort. This is deviating from the data published by Kourelis et al., where also a strict classification for cardiac and renal patients was chosen, but IGLV6-57 was identified as the most dominant family for patients with renal involvement [10].

We were able to detect IGLV3-21 as the most dominant IGLV-family for patients with cardiac involvement and IGLV1-44 for patients with dominant kidney involvement. In 2012, Perfetti et al. detected IGLV1-44 as the most frequent and IGLV3-21 as the second most frequent IGLV-family for dominant heart disease [9].

Another new aspect of our study is, that we examined not only the IGLV-segment, but the composition of the entire LC in more detail. We were able to make statements about the possible connections of the IGLJ and IGLC-segments.

Surprisingly, for IGLJ3 a joining between IGLC2 and IGLC3 was found. Due to the genomic loci and the arrangement in IGLJ-IGLC cassettes [27] a connection between IGLJ3 and IGLC2 seems to be impossible, but based on the sequencing data the segments were assigned properly. This finding might be an artefact produced by false identification of the respective IGLJ-family through a high mutation rate, because IGLJ2 and IGLJ3 sequences only differ by three cDNA nucleotides. In several cases, a connection between the IGLJ3-segment with IGLC2-segment without a mutation in the IGLJ-segment was found in this study. A triple mutation of IGLJ2 towards a “IGLJ3 like” sequence is assumed unlikely–even not impossible. Also, Ensembl and dbSNP data does not suggest frequent occurrence of this multi nucleotide variation. Vice versa, also the cDNA sequence of IGLC2 and IGLC3 differ by three nucleotides which however are 53 and 34 base pairs apart from each other even thought, only by one AS coded by the third nucleotide. The corresponding sequences in this study show at the first position in all cases the nucleotide corresponding to the IGLC2-segment, at the second position show only 3/12 sequences the corresponding nucleotide and at the third position 11 sequences show the corresponding nucleotide, in one case this position is not covered. This third position lead to the only differing AS-position between the IGLC reference sequences. So also, a mutation towards a “IGLC2 like” sequence is possible but is not a satisfying solution especially these sequences showed no other mutations in this segment. The assignment of IGLC2 (by AS sequences) was fully supported by cDNA in three respective cases: all three IGLC2/3 differentiating nucleotide positions would have to be mutated independently toward IGLC3 for false identification. In another six cases two independent mutations would have had to occur. As the 8/12 cDNA sequences in question showed no blurring or overlapping signals so a major impact of clonal subtypes on the cDNA sequence is not suspected. Even in the used CD138 positive fraction of the AL BM samples the clonal fraction has to be quite large to impact sanger sequencing results and should have been picked up in the mutational hotspots of CDR1, 2 and 3 as blurring or overlapping signals. As it was found in this study, were 18 of the 126 IGLJ3*02 AL LC-sequences in the AL-Base database [25] assigned to a linkage with IGLC2. This finding might point to an interesting locus for future investigation.

Finally, we also investigated the connection of the IGLV-segments with IGLJ and IGLC. For the IGLV1-family, including IGLV1-40, IGLV1-44, IGLV1-47 and IGLV1-51 there was a linkage with IGLJ1 and IGLC1 as well as linkages with IGLJ2/IGLJ3 and IGLC2/IGLC3 detected. This was also shown for the IGLV2-family, including IGLV2-8 and IGLV2-14. In contrast, linkage of IGLJ1 and IGLC1 with IGLV6-57 could not be determined. Interestingly, for the most dominant IGLV-families for dominant heart and dominant heart and kidney involvement IGLV3-21 and IGLV6-57 a linkage to IGLJ2 and IGLC2 was detected most frequent. No uniform statement can be made for the subgroups of the IGLV3-family. For IGLV3-21, IGLV3-19, as well as IGLV3-25, no linkage with IGLJ1 and IGLC1 was detected. Interestingly for IGLV3-1 a linkage with IGLJ1 and IGLC1 could be detected in 75% of the cases. These results suggest that spatial genetic proximity to the IGLJ and IGLC loci may be crucial. The IGLV3-1-locus is located on chromosome 22q11.22 closest to IGLJ1 and IGLV6-57 furthest away. Therefore, our results can give an indication of the possible linkage patterns, but due to the small sample number of individual IGLV-families further investigation are needed to confirm our findings.

In conclusion, our method of CD138+ cell sorting not only allows us to achieve a very high purity of the sample, but also provides information on the clonality of the sample via FISH analysis. We were able to confirm a specific enrichment of IGLV-families in relation to organ tropism in systemic AL amyloidosis. We detected IGLV3-21 as the most dominant IGLV-family for dominant heart involvement (28%), IGLV1-44 for dominant kidney involvement (20%) and IGLV6-57 for dominant heart and kidney involvement (46%). In addition, for the first time we took a closer look at the complete LC and the linking of IGLV, IGLJ and IGLC segments. Here, we were able to identify dominant linkage pattern between IGLJ and IGLC. IGLJ1 was found exclusively linked to IGLC1 and IGLJ2 to IGLC2. For IGLJ3 a joining with IGLC2 and IGLC3 could be observed. In the linkage of IGLV-segments with IGLJ and IGLC, a certain genetic proximity seems to play a decisive role.

## Supporting information

S1 Fig Overview of the inclusion of patients in this study. (TIF)

S2 Fig Comparison of the dFLC values at diagnosis and the plasmacell-infiltration. (TIF)

S3 Fig Comparison of the percentage of the main clone based on the FISH results in comparison. The highest measured percentage of a genetic aberration in the FISH result was defined as the percentage of the main clone.
(TIF)

S4 Fig Comparison of the dFLC values between the IGLV-subfamilies. All patients are included regardless of the organ manifestation. The dFLC values were collected at diagnosis.
(TIF)

S1 Table Oligonucleotides. (DOCX)

S2 Table Raw data IGLV-family germline distribution. HK = dominant heart and kidney involvement, H = dominant heart involvement, K = dominant kidney involvement.
(DOCX)

S3 Table Raw data IGLV-subfamily germline distribution. HK = dominant heart and kidney involvement, H = dominant heart involvement, K = dominant kidney involvement.
(DOCX)
