# Supplementary figures and images for: Analysis of the complete lambda light chain germline usage in patients with AL amyloidosis and dominant heart or kidney involvement
Source: PLoS One. 2022 Feb 25;17(2):e0264407. doi: 10.1371/journal.pone.0264407 (PMC8880859; doi:10.1371/journal.pone.0264407)

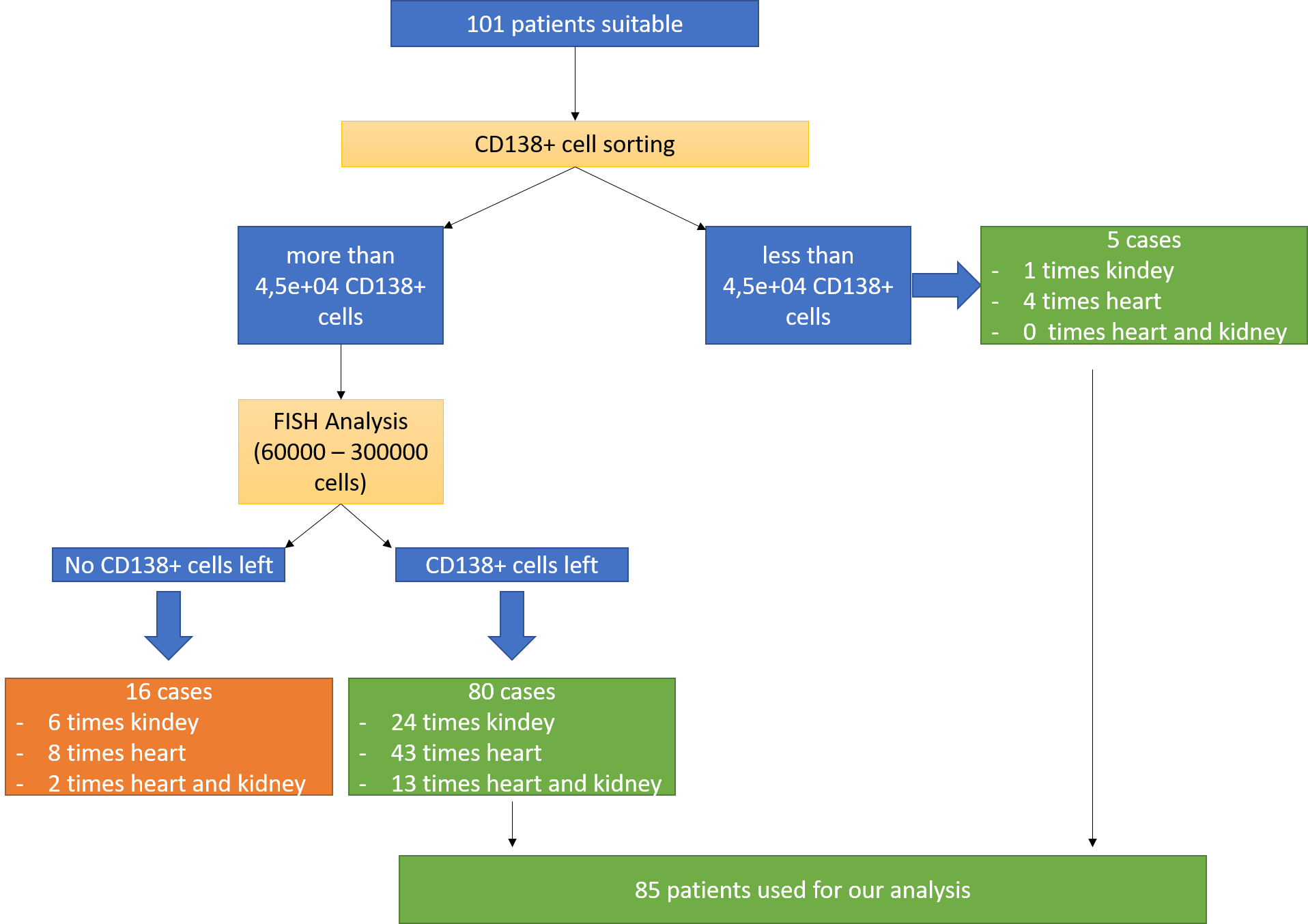

Supplement: S1 Fig — (TIF) [file pone.0264407.s001.tif]

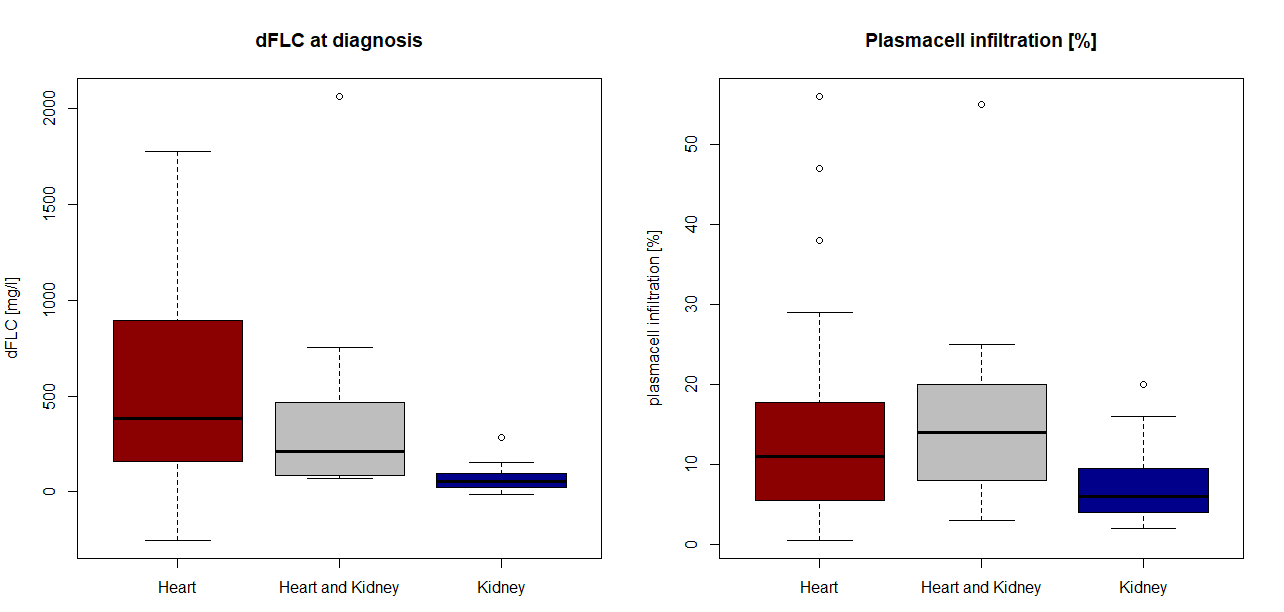

Supplement: S2 Fig — (TIF) [file pone.0264407.s002.tif]

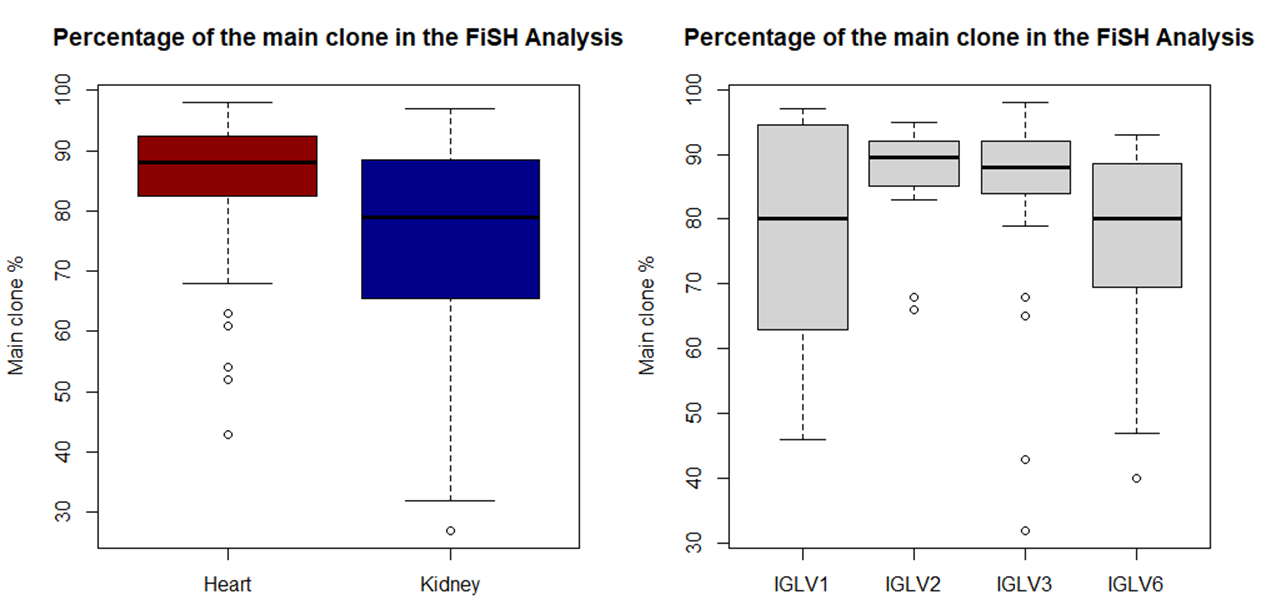

Supplement: S3 Fig — The highest measured percentage of a genetic aberration in the FISH result was defined as the percentage of the main clone. (TIF) [file pone.0264407.s003.tif]

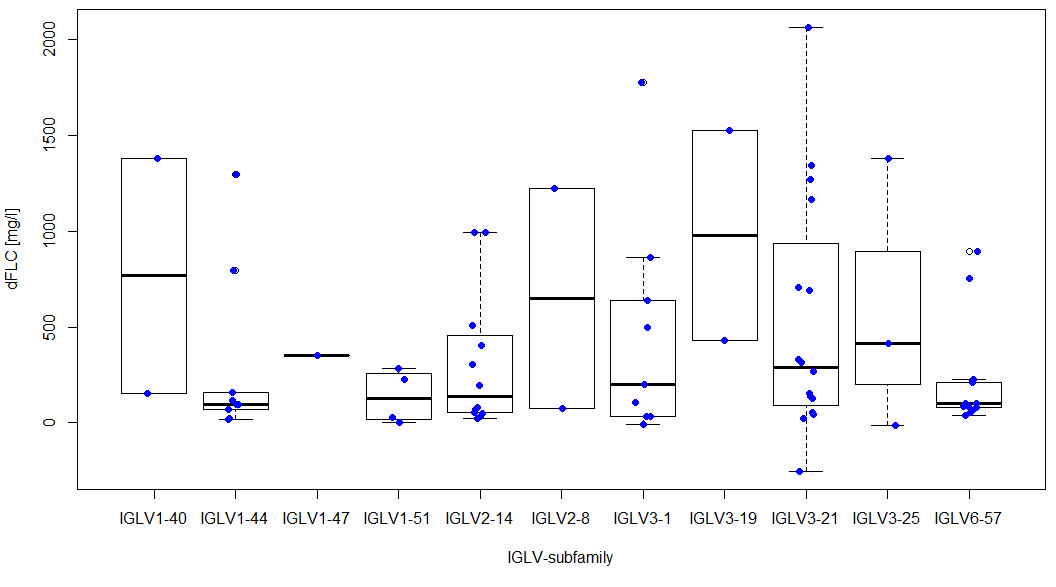

Supplement: S4 Fig — All patients are included regardless of the organ manifestation. The dFLC values were collected at diagnosis. (TIF) [file pone.0264407.s004.tif]
