# Supplementary material for: Analysis of the complete lambda light chain germline usage in patients with AL amyloidosis and dominant heart or kidney involvement
Source: PLoS One. 2022 Feb 25;17(2):e0264407. doi: 10.1371/journal.pone.0264407 (PMC8880859; doi:10.1371/journal.pone.0264407)
Supplement: S1 Table — (DOCX) [file pone.0264407.s005.docx]

| **Name** | **Sequence** |
| --- | --- |
| CLKL_A_rv_NB | CACTGTCTTCTCCACGGTG |
| VLKL3_A_fw_NB | CCTATGAGCTGACACAGCC |
| VLKL6_A_fw_NB | CAGCCCCACTCTGTGTCG |
