# Supplementary material for: Analysis of the complete lambda light chain germline usage in patients with AL amyloidosis and dominant heart or kidney involvement
Source: PLoS One. 2022 Feb 25;17(2):e0264407. doi: 10.1371/journal.pone.0264407 (PMC8880859; doi:10.1371/journal.pone.0264407)
Supplement: S3 Table — HK = dominant heart and kidney involvement, H = dominant heart involvement, K = dominant kidney involvement. (DOCX) [file pone.0264407.s007.docx]

|  | IGLV  1-40 | IGLV  1-44 | IGLV  1-47 | IGLV  1-51 | IGLV  2-8 | IGLV  2-14 | IGLV  3-1 | IGLV  3-19 | IGLV  3-21 | IGLV  3-25 | IGLV  6-57 | NA |
| --- | --- | --- | --- | --- | --- | --- | --- | --- | --- | --- | --- | --- |
| HK [n] | 0 | 0 | 1 | 1 | 1 | 1 | 0 | 0 | 1 | 0 | 6 | 2 |
| H [n] | 1 | 5 | 0 | 0 | 1 | 8 | 6 | 2 | 13 | 2 | 4 | 5 |
| K [n] | 1 | 5 | 0 | 3 | 0 | 4 | 3 | 0 | 3 | 1 | 3 | 2 |
